# Soluble ST2 in Heart Failure: A Clinical Role beyond B-Type Natriuretic Peptide

**DOI:** 10.3390/jcdd10110468

**Published:** 2023-11-17

**Authors:** Mauro Riccardi, Peder L. Myhre, Thomas A. Zelniker, Marco Metra, James L. Januzzi, Riccardo M. Inciardi

**Affiliations:** 1 Institute of Cardiology, ASST Spedali Civili di Brescia, Department of Medical and Surgical Specialties, Radiological Sciences, and Public Health, University of Brescia, 25121 Brescia, Italy; mauro94rc@hotmail.it (M.R.); metramarco@libero.it (M.M.); 2 Department of Cardiology, Division of Medicine, Akershus University Hospital, Lørenskog, 1478 Nordbyhagen, Norway; p.l.myhre@medisin.uio.no; 3 K.G. Jebsen Center for Cardiac Biomarkers, Institute of Clinical Medicine, University of Oslo, 0313 Oslo, Norway; 4 Department of Internal Medicine II, Division of Cardiology, Center of Cardiovascular Medicine, Medical University of Vienna, 1090 Vienna, Austria; thomas.zelniker@meduniwien.ac.at; 5 Cardiology Division, Massachusetts General Hospital, Harvard Medical School, and Baim Institute for Clinical Research, Boston, MA 02215, USA; jjanuzzi@mgb.org

**Keywords:** heart failure, solubleST2, natriuretic peptides

## Abstract

Soluble (s)ST2 has been proposed as a useful biomarker for heart failure (HF) patient management. Myocardial damage or mechanical stress stimulate sST2 release. ST2 competes with a membrane bound receptor (ST2 ligand, or ST2L) for interleukin-33 (IL-33) binding, inhibiting the effects induced by the ST2L/IL-33 interaction so that excessive sST2 may contribute to myocardial fibrosis and ventricular remodeling. Compared to natriuretic peptides (NPs), sST2 concentration is not substantially affected by age, sex, body mass index, kidney function, atrial fibrillation, anemia, or HF etiology, and has low intra-individual variation. Its prognostic role as an independent marker is well reported in the literature. However, there is a gap on its use in combination with NPs, currently the only biomarkers recommended by European and American guidelines for HF management. Reflecting the activation of two distinct biological systems, a benefit from the use of sST2 and NP in combination is advocated. The aim of this review is to report the current scientific knowledge on sST2 in the acute and chronic HF settings with a particular attention to its additive role to natriuretic peptides (NPs).

## 1. Introduction

Quantifying concentrations of circulating biomarkers plays a major role in most cardiovascular (CV) diseases, including heart failure (HF) [1].

An ideal biomarker in HF should be (1) measured non-invasively and at low cost, (2) highly sensitive to allow for the early detection of the disease, (3) unaffected or minimally affected by comorbid conditions, and (4) responsive to treatment effects [2]. The most established biomarkers in HF are B-type natriuretic peptide (BNP) and its co-secreted amino-terminal pro-peptide fragment (NT-proBNP), which reflect cardiac trans-mural wall stress. BNPs are strong predictors of HF presence and severity and provide prognostic information; therefore, BNP and NT-proBNP have a class 1 recommendation in the current European Society of Cardiology (ESC) and American College of Cardiology/American Heart Association (ACC/AHA) HF guidelines for these indications [3,4].

Beyond their well-established diagnostic role in acute and chronic setting, the role of BNP and NT-proBNP in risk stratification is gaining more momentum in clinical practice. In fact, low values of NPs at discharge reflect the achievement of greater decongestion, which correlate with a lower risk of re-hospitalization and death. In addition, the pre-discharge value can be used to determine the intensity of monitoring and the timing for follow-up visits [5].

However, there are important limitations to natriuretic peptide (NP) testing in HF. Most important is the impact caused by conditions commonly associated with HF such as atrial fibrillation (AF), kidney dysfunction and obesity, as well as a wide range of cardiac and non-cardiac abnormalities associated with an increase in parietal tension without necessarily being linked to fluid retention [5]. NP concentrations also vary substantially with age and sex, which introduces difficulties in using thresholds for decision making. Beyond these issues, the concentrations of BNP and NT-proBNP only reflect one aspect of the considerably complex pathophysiology of HF. Accordingly, a broader palette of biomarkers would be expected to provide an important depth of understanding of individuals affected by the diagnosis.

Numerous other biomarkers have been evaluated in HF and are under investigation. Some of these have been more convincing and are used in some clinics today. Of particular prominence is soluble ST2 (sST2) [1], which was first classified as an indicator of ventricular myocyte stress [6], but is mainly produced in extracardiac tissues [7] in response to inflammatory and fibrotic stimuli [8], representing an indicator of the myocardial fibrotic process and a predictor of cardiac remodeling [9,10,11]. 

The aim of this review is to update the current knowledge on sST2 in acute and chronic HF with a particular attention to its additive role to NPs.

## 2. sST2 Biology

ST2 is a member of the interleukin (IL)-1 receptor family [12], whose gene is located on human chromosome 2q12. Alternative promoter splicing and 3′ processing of the mRNA are responsible for the production of two different forms: a soluble receptor, named sST2; or a transmembrane receptor, named ST2L [2,8,13]. ST2 was first described in 1989 [14,15]. 

The literature mistakenly called ST2 a “suppressor of tumorigenicity 2”, when, in fact, the original name it was given was “growth stimulation expressed gene 2”, then renamed “serum stimulation-2”, as it was first discovered to function as a mediator of type 2 inflammatory responses [16].

Its role as a cardiac marker was suggested in 2002 by Weinberg et al. [17], analyzing the expression of 7.000 genes in cardiomyocytes undergoing mechanical strain and noting that myocardial transcripts of ST2 increased significantly in response to this stimulus. This is curious and important, as the main source of sST2 in the circulation in patients with HF does not appear to be the heart. Indeed, it has been shown that type 2 pneumocytes represent a relevant source of sST2 in HF patients and concentrations of sST2 in pulmonary edema from individuals with HF are strongly correlated to blood values [7]. This link to pulmonary pathophysiology may explain why sST2 correlates with the presence and severity of pulmonary congestion in HF [18]. This is in contrast to NPs, which are also upregulated in HF and correlate with pulmonary congestion, but are only expressed in cardiomyocytes and not the lungs. For this reason, an additional role of sST2 relative to NPs for the evaluation of the HF phenotype and prognosis seems likely from a biological perspective.

**Figure 1:**
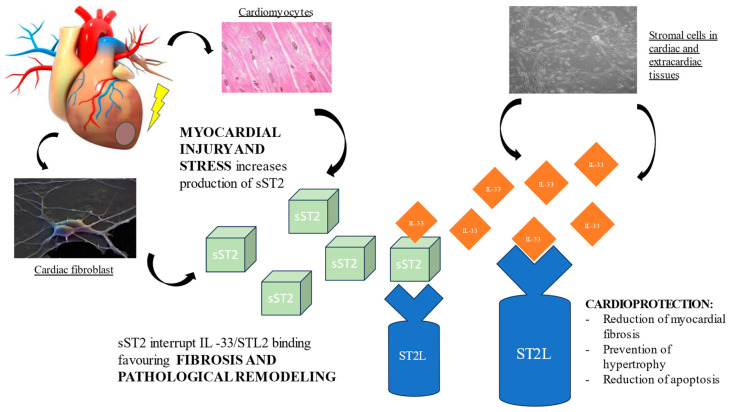
Pathological role of sST2 in promoting fibrosis and ventricular remodeling.

The cognate ligand of ST2 is interleukin-33 (IL-33), a cardiac fibroblast protein released by stromal cells in cardiac and extracardiac tissues. Depending on co-stimulatory factors, IL-33 can act either as a pro- or anti-inflammatory cytokine. At the cardiac level, the ST2L/IL-33 interaction initiates a complex cardioprotective biochemical cascade, which prevents cardiomyocyte hypertrophy, apoptosis, and myocardial fibrosis, thereby improving cardiac function. However, when the heart is subjected to damage or mechanical stress, cardiomyocytes and cardiac fibroblasts secrete sST2, which, competing with ST2L for the IL-33 binding site, antagonizes the cardioprotective effect, contributing to myocardial fibrosis and ventricular remodeling [12,19,20]. (Figure 1) Hence, the activation of the ST2L/IL-33 pathway is a beneficial adaptive response in cardiac disease, which is offset by sST2 secretion.

The “inflammatory hypothesis” of atherosclerosis implies that the presence of inflammation favors the formation, growth, and, finally, the instability of atherosclerotic plaques, favoring the onset of cardiovascular events [21]. The IL-33-ST2L pathway could inhibit the development of atherosclerosis through the immune response toward a T helper 2, macrophage 2 phenotype, while high sST2 values could promote plaque development, sequestering IL-33 [22]. As a result, in patients with non-ST-elevation acute coronary syndrome, the level of serum sST2 might be a useful predictive marker of plaque vulnerability [23]. From a neurological point of view, sST2 levels increase in patients with mild cognitive impairment, suggesting that impaired IL-33/ST2 signaling may contribute to the pathogenesis of Alzheimer’s disease [24] and an elevation in sST2 serum concentration represents and tracks disease progression. 

sST2 also appears to be involved in the pathogenesis of cancers, trying to counterbalance the tumorigenesis effect of IL-33/ST2, and could therefore be used for non-invasive diagnostic tests, as a prognostic marker and for treatment monitoring [25]. For example, in gastric cancer, sST2 was significantly associated with a more advanced tumor stage (*p* = 0.018), metastatic disease (*p* = 0.014), and was significantly correlated with the duration of the disease (*p* = 0.0017) [26]. Similarly, serum levels of IL-33 and sST2 were significantly higher in breast cancer patients in comparison with healthy volunteers [25,27].

ST2L is a cell-surface marker of T helper type 2 (Th2) lymphocytes and, therefore, IL-33/ST2 has an essential role in immune regulation. As a result, it has been associated with diseases characterized by a predominantly Th2 response, such as asthma, pulmonary fibrosis, rheumatoid arthritis, collagen vascular diseases, sepsis, trauma, fibroproliferative diseases and ulcerative colitis [25]. IL-33/ST2 also has a profibrotic role in the pathogenesis of hepatic diseases. In this regard, sST2 has an opposite function, and its elevation in liver cirrhosis, hepatocellular carcinoma and hepatitis B infection could be a sign of a positive regulatory loop in the remission of these diseases [28,29].

## 3. sST2 Prognostic Role

Being a non-cardiac-specific biomarker, sST2 is less useful for diagnosing HF, but has proven helpful for risk stratification, both in chronic and acute settings [30]. 

### 3.1. Incident HF

The vast majority of studies considered sST2 in the context of HF, whereas more limited and discordant data were published regarding sST2 concentrations in healthy individuals or at risk for developing HF (Table 1). It has been shown in a sample of individuals without HF that higher levels are associated with male sex, older age (in women), increased aortic stiffness and, consequently, increased systolic blood pressure (more notably in men), the use of antihypertensive medication, and diabetes, all factors related to the development of HF [31,32]. In the Framingham Heart Study, which included 3428 participants, sST2 was associated with a higher risk of developing HF (Hazard Ratio (HR) per 1 standard deviation (SD) 1.45; 95% CI 1.23–1.70; *p* < 0.001) [33]. This study was the first to examine the prognostic value of sST2 measurements in the general population, showing that higher levels of circulating sST2 can be detected in apparently healthy individuals and precede adverse outcomes. Similarly, in adults with mild to moderate chronic kidney disease at entry in the CRIC (Chronic Renal Insufficiency Cohort) study, sST2 levels were statistically related to the risk of developing HF (HR per 1 SD 1.19; 95% CI, 1.05–1.35), in particular with preserved ejection fraction (HR per 1 SD 1.27; 95% CI, 1.07–1.51) [34]. Furthermore, sST2 has been shown to improve risk stratification after myocardial infarction, and to significantly improve the survival prediction beyond that of GRACE (Global Registry of Acute Coronary Events) and TIMI (Thrombolysis in Myocardial Infarction) scores [35]. Higher values of sST2 also independently predict incident HF following a myocardial infarction [36]. While an sST2 measurement may already be indicative in a healthy population, the prognostic information provided by serial sST2 measurements appears to be even more relevant than BNPs values for predicting major adverse CV events (MACEs) and to have an additive role. In fact, in 282 patients with CV risk factors at risk of developing MACE within the STOP HF cohort, a one-unit increase in sST2 variation corresponded to an increase of about 8% in the rate of one or more MACE [37]. 

It is, however, important to highlight studies with neutral results. In a healthy general population from Finland including 8444 men and women, sST2 did not improve the long-term prediction of CV events including HF (HR per 1 SD of log sST2 1.06; 95% CI 0.96 to 1.17) [38]. Similarly, in an analysis performed using data from four community-based cohorts with 12.5 years of follow-up, sST2 levels were not significantly associated with incidental HF in either women or men (HR per 1 SD in women 1.12; 95% CI 1.02–1.22; HR in men 1.08; 95% CI 1.02–1.22; *p* for interaction 0.40), even after adjusting for NPs levels (HR 1.07; 95% CI 0.97–1.18; *p* = 0.157; HR 1.01; 95% CI 0.91–1.11; *p* = 0.857, respectively) [39]. More recently, sST2 was not predictive of future development of new onset HFpEF in a retrospective analysis of a longitudinal STOP-HF study of thirty patients [40]. 

### 3.2. Acute HF

In acute HF (AHF), increased levels of sST2 appear to be linked to the peripheral release of pro-inflammatory cytokines by vascular endothelial cells and lung tissue in response to congestion and inflammation [7,41,42]. As a result, a higher concentration of sST2 is associated with more severe pulmonary congestion in AHF [7]. It also positively correlates with echocardiographic measures of right ventricular dysfunction and increased central venous pressure [43]. Finally, it has recently been identified as a surrogate marker of poor diuretic response in patients with AHF and kidney dysfunction [44]. 

Currently, in this setting, the sST2 diagnostic value is controversial and merits further studies. Henry-Okafor et al. demonstrated among patients presented to the emergency department with signs or symptoms of AHF that sST2 was not significantly associated with the diagnosis of AHF in adjusted models (*p* = 0.33). The area under the curve (AUC) for sST2 was 0.62 (95% confidence interval [CI] = 0.56–0.69), suggesting moderately low diagnostic utility [45]. In contrast, the Pro-Brain Natriuretic Peptide Investigation of dyspnea in the Emergency Department (PRIDE) study [46] analyzed 593 patients who were admitted to the emergency department for acute dyspnea independent of the presence of HF. Using an assay no longer utilized for measuring sST2, the concentrations of the biomarker were significantly higher in acute decompensated HF (ADHF) patients than in non-HF patients (1.08 vs. 0.18 ng/mL; *p* < 0.001) [46]. 

In contrast with the diagnostic role, the prognostic role of sST2 has been described in several studies (Table 2). In the PRIDE study [46], an sST2 concentration ≥20 ng/mL strongly predicted death at 1 year in dyspneic patients as a whole (HR 5.6, 95% CI 2.2–14.2; *p* < 0.001) as well as in those with AHF (HR 9.3, 95% CI 1.3–17.8; *p* = 0.03). In another study of 1528 ADHF patients enrolled from the HF Center of Beijing Fuwai Hospital, sST2 concentrations were measured within 12 h of hospitalization for HF [47]. The concentrations of sST2 were significantly higher among patients with adverse events (AEs), defined as all-cause death and cardiac transplantation, in comparison to patients without AEs (33.6% vs. 55.6%, *p* < 0.001). Patients in the fourth quartile of sST2 as measured with a high-sensitivity enzyme-linked immunosorbent assay (>55.6 ng/mL) had a higher rate of AEs if compared with patients with the lowest sST2 concentration quartile (≤25.2 ng/mL) (HR 6.92, 95% CI 4.71–10.16; *p* < 0.001), with a graded increase in AEs’ rates at 3 months, 1 year and 3 years according to sST2 quartiles. Cox regression showed that sST2 concentrations were significantly associated with the combined endpoint in univariable and multivariable analysis after adjustment for several variables, including NT-proBNP levels (per 1 log unit, adjusted HR 1.52, 95% CI: 1.30–1.78; *p* < 0.001). Similarly, Pascual-Figal et al. [48] conducted a prospective study on 107 ADHF inpatients and demonstrated that patients who died had significantly higher concentrations of sST2 (HR per 10 ng/mL 1.09, 95% CI 1.03–1.15, *p* = 0.005 in multivariable analysis). Using ROC analysis, the optimal cut-off points for the prediction of death were >65 ng/mL.

Lastly, in the Acute Study of Clinical Effectiveness of Nesiritide in Decompensated Heart Failure (ASCEND-HF) trial [49], sST2 was measured at 48–72 h after hospital admission and again after 30 days in 858 AHF patients. Higher sST2 levels were associated with an increased mortality risk at 180 days (*p* < 0.001), although this association was not significant after adjustments for NT-proBNP and the ASCEND-HF risk model. Subjects with persistently high (>60 ng/mL) sST2 levels at 30 days had higher 180-day death rates than those with lower sST2 levels (adjusted HR 2.91, *p* = 0.004). 

Serial sST2 measurements during hospitalization are also recommended because they may provide a basis for enhanced clinical decision making and improve the accuracy of mortality prediction [62]. In fact, in 207 patients with AHF, sST2 decreased significantly during the first 48 h (median decrease 33%). The decrease was less pronounced in non-survivors compared with survivors (median −25% vs. −42%, respectively; *p* < 0.01) and early ST2 changes independently predicted 1-year mortality (HR 1.07 for every increase of 10%; *p* = 0.02) [50]. Similarly, Boisot et al. have shown that from admission to discharge, the percent change in sST2 was strongly predictive of 90-day mortality: those patients whose sST2 values decreased by 15.5% or more during the study period had a 7% chance of death, whereas patients whose sST2 levels failed to decrease by 15.5% in this time interval had a 33% chance of dying [51].

sST2 concentration tends to decrease after the initiation of HF treatment and from decongestion as a result of reducing the inflammatory picture, reducing the cardiotoxic mechanism and reducing mechanical stress and volemia [50]. Thus, changes in sST2 concentration during the up-titration of HF therapies and diuresis may provide important insights into therapeutic response, helping the physician in determining both the correct therapeutic strategy and prognosis. However, with regard to the first scenario, in the STADE-HF (sST2 As a help for management of Diagnosis, Evaluation and management of HF) trial [63], the use of sST2 to guide therapy at day 4 after admission in 123 patients with AHF did not reduce readmissions at 1 month (10% in the usual care arm vs. 32% in the sST2 group, *p* = 0.11). Therefore, currently, the use of sST2 is not recommended to guide medical therapy. As for the prognostic role, in ADHF, the current evidence recommends that ST2 concentration should be assessed at baseline (for initial risk assessment) and after treatment to reflect therapeutic effectiveness, independent of repeated measurements of NPs [62,64,65]. In particular, the percent of change in sST2 concentrations during AHF treatment appears to predict 90-day mortality regardless of BNP or NT-proBNP levels [51].

The prognostic importance of sST2 in AHF was confirmed in a meta-analysis [52] of 4835 patients with AHF, which concluded that both admission and discharge sST2 were predictive of all-cause death (HR per log unit increase 2.46, 95% CI 1.80–3.37 and HR 2.06, 95% CI 1.37–3.11, respectively) and CV death (HR per log unit increase 2.29, 95% CI 1.41–3.73, and HR 2.20, 95% CI 1.48–3.25, respectively), and that discharge sST2 predicted rehospitalization for HF (HR per log unit increase 1.54, 95% CI 1.03–2.32).

Based on the International ST2 Consensus Panel published in 2015, an sST2 ≥ 35 ng/mL value was recommended as a predictor threshold for a poor prognosis in AHF [66]. However, this cut-off may be too sensitive (and not specific enough) for AHF, and a higher cut-off (up to 65 ng/mL) has been suggested [67].

Of note, the prognostic role of sST2 is consistent in both HF with reduced EF (HFrEF) and HF with preserved EF (HFpEF) patients, as demonstrated by Manzano-Fernández et al. [53], who prospectively enrolled 447 patients with AHF. The sST2 concentrations were greater in patients with HFrEF (*n* = 250) than in those with HFpEF (*n* = 197) but elevated sST2 concentrations were associated with a greater mortality risk in both populations, even after adjusting potential confounders and NT-proBNP. Positive results in patients with HFpEF have also been shown more recently by Sugano et al. [54] and Shah et al. [55]. 

### 3.3. Chronic HF

Much as with AHF, the concentrations of sST2 represent a strong prognostic measure in chronic HF (CHF) (Table 2). For example, in a multicenter prospective cohort study that included 1141 CHF outpatients, sST2 > 36.3 ng/mL predicted a higher risk of AEs (death or transplantation), demonstrating that sST2 is a powerful prognostic biomarker in CHF [56]. In another large cohort of patients with CHF (*n* = 4268), a single sST2 measurement yielded a prognostic value independent of age, HF etiology, LVEF, estimated glomerular filtration rate (eGFR), and the concentrations of other CV biomarkers, including NT-proBNP [57]. Similarly, Emdin et al. found that the risk of all-cause and CV deaths, and HF hospitalization increased by 26%, 25%, and 30%, respectively, per each doubling of sST2 [58]. In 2331 patients with CHF and LVEF < 35% in the US HF-ACTION study [59], sST2 was significantly associated with CV mortality and HF hospitalization, as well as with all-cause mortality, even after accounting for confounders including NT-proBNP. In the CORONA study of 1449 patients with ischemic CHF and LVEF <40%, sST2 was associated with CV death, non-fatal myocardial infarction, and stroke, but the association was attenuated after adjustment for NT-proBNP [60]. 

Several studies also provide evidence of a significant association between increased sST2 levels and outcome in HFpEF [61,68,69]. Guideline-directed medical pharmacotherapies of CHF seem to reduce sST2 levels and have been proven for β-blockers and sacubitril/valsartan [70]. For this reason, sST2 may have a role in monitoring the response to pharmacological treatment in the chronic setting. 

## 4. sST2 Assessment in Addition to NPs: Is There a Role?

As previously mentioned, NPs have important limitations as they are affected by common comorbidities in patients with HF. AF and renal disease substantially increase the concentrations of NPs, while obesity is associated with lower NP levels [71]. In addition, NPs are mainly expressed from the LV with its cardiomyocyte dominance, risking underestimating both systemic fluid overload and right-sided HF [5]. Finally, studies examining the role of therapy with a goal of NP suppression have not demonstrated lower event rates associated with this approach [72,73]. 

Because sST2 elevations reflect the activation of distinct biological systems compared with the NPs, there is a knowledge gap as to whether sST2 can provide additional prognostic information [53,59]. Multiple studies have shown only a moderate correlation between sST2 and NT-proBNP [56], confirming that these two markers assess different aspects of the HF syndrome. Moreover, sST2 values are not directly influenced by age, sex, BMI, kidney function, AF, anemia, or HF etiology and, compared to other CV biomarkers, these have a low intra-individual variation [74,75,76]. There is evidence of better prognostic stratification in the combination of the two markers in both AHF and CHF (Table 3). In 593 dyspneic patients with and without AHF presenting to an emergency department, the combination of sST2 and NT-proBNP more accurately predicted death (AUC 0.80, 95% CI 0.76–0.84) than the single biomarker assessment, and elevation in both markers was associated with the highest rates of death at 1 year in the entire patient cohort, as well as in AHF [46]. Similarly, Zhang et al. [47] showed that baseline sST2 concentrations appeared to have a more pronounced positive predictive value when compared with those of NT-proBNP, which might indicate the added prognostic value of sST2 compared to NT-proBNP. Lastly, in the ASCEND trial [49], adding 48 to 72 h of follow-up sST2 to the ASCEND-HF risk model, plus follow-up NT-proBNP, correctly reclassified 15.6% of subjects for the 180-day death endpoint. The combination of these two markers has clinical value regardless of the LVEF. Among patients discharged after hospitalization for AHF, the combination of sST2 levels with BNP levels added prognostic information with a significant 7-fold increase in the risk of worse events for both biomarkers elevated in HFrEF and 5-fold increase in risk in HFpEF [77]. 

**Figure 2:**
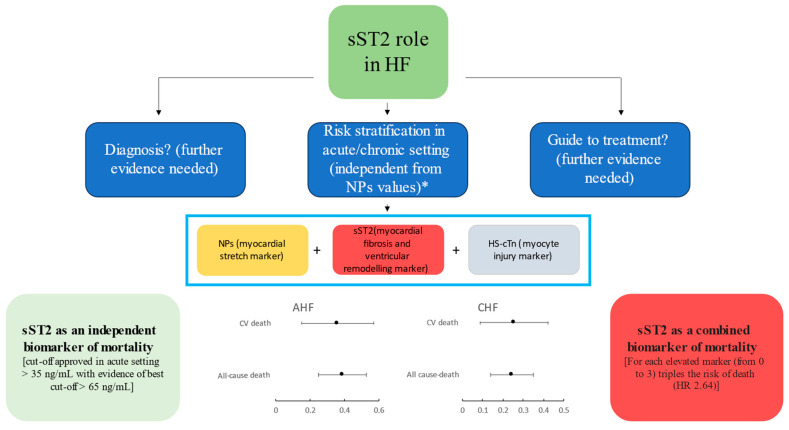
Current evidence on the role of sST2 in HF patients. * Not directly influenced by age, sex, BMI, kidney function, AF, anemia or HF etiology, and it has a low intra-individual variation. Forest plot analysis showing hazard ratios and 95% confidence interval of sST2 and mortality in acute and chronic HF settings (adapted from J Am Coll Cardiol HF 2017; 5:280–6; and J Am Coll Cardiol HF 2017; 5:287–96) [52,81].

Prognostic stratification in AHF can be further implemented through the addition of other biomarkers to sST2 and NPs, including high-sensitive cardiac troponin-T (hs-cTnT) (Figure 2). In fact, the presence of all three biomarkers below their optimal cut-off at presentation was associated with an absence of mortality during follow-up, whereas about half of patients with such elevated markers died [48]. The risk of death triples for each elevated marker (from 0 to 3) in adjusted analysis (for each elevated marker, HR 2.64, 95% CI 1.63–4.28, *p* < 0.001). For this reason, this triad of biomarkers, reflecting different facets of HF pathophysiology, has been included in the Barcelona Bio-HF calculator, a risk score that calculates the risk of all-cause death and/or HF hospitalization [80]. 

Even among patients with CHF, the combined assessment of sST2 and NT-proBNP is more effective in identifying a high-risk subgroup than the individual assessment of both biomarkers [47,56]. Similarly, Bayes-Genis et al. [78] showed that among ambulatory patients with CHF, those with elevated levels of both sST2 and NT-proBNP had a markedly increased risk (HR 6.38, 95% CI 4.67–9.25, *p* < 0.001), again indicating that the assessment of both sST2 and NT-proBNP is more effective in identifying a high-risk subgroup than individual assessments of either biomarker. Lastly, a post hoc analysis of the MUSIC (MUerte Súbita en Insuficiencia Cardìaca) study [79] analyzed the role of sST2 for the prediction of sudden cardiac death (SCD) in patients with mild-to-moderate HF and LV systolic dysfunction. In this study, the presence of both elevated NT-proBNP and sST2 (Odds Ratio 37.3, 95% CI 4.0–350; *p* = 0.002) was more predictive of SCD than evaluating each biomarker separately. This combined variable added incremental prognostic value to the multivariable regression model. Only 4% of patients experienced SCD for neither sST2 nor NT-proBNP above the ROC-derived cut-off points; 34% for either of the biomarkers above; and 71% for both biomarkers above.

## 5. Conclusions

sST2 is a strong independent prognostic marker in HF patients regardless of LVEF and NPs concentrations. Due to its secretion following independent pathways, its role appears useful to improve risk stratification beyond NPs. Up to now, however, the available evidence has come from non-randomized studies, thus not enabling any mention of it in recent ESC3 and ACC/AHA4 guidelines on HF management, while the 2013 ACC/AHA83 clinical practice guidelines gave a Class IIb recommendation for sST2 measurements only in CHF for the purpose of risk stratification and prognostication. Further studies are therefore needed in order to frame this promising biomarker in the management of HF.

## Figures and Tables

**Table 1:** Association between sST2 levels and incident HF.

Author	Patients	Type of Cohort	Results
Wang et al. [33]	3428	Asymptomatic community-based population	sST2 was associated with the risk of developing HF (HR per 1 SD 1.45; 95% CI 1.23–1.70; *p* < 0.001).
Bansal et al. [34]	3314	CKD population	sST2 was associated with the risk of developing HF (HR per 1 SD 1.19; 95% CI, 1.05–1.35), in particular with preserved ejection fraction (HR = 1.27; 95% CI, 1.07–1.51).
Watson et al. [37]	282	Asymptomatic community-based population	The sST2 increase from baseline to follow up led to an increased risk of incident MACE by approximately 7.9%.
Hughes et al. [38]	844	Asymptomatic community-based population	sST2 did not improve long-term prediction of CV event, including HF (HR per 1 SD 1.06; 95% CI = 0.96–1.17).
Suthahar et al. [39]	22,756	Asymptomatic community-based population	sST2 levels were not significantly associated with incident HF in either women or men (HR per 1 SD in women 1.12; 95% CI 1.02–1.22; HR in men 1.08; 95% CI 1.02–1.22; *p* for interaction 0.40).

CKD, chronic kidney disease; CI, confidence interval; HF, heart failure; HR, hazard ratio; MACE, major adverse cardiovascular event; SD, standard deviation; CV, cardiovascular.

**Table 2:** Evidence of prognostic role of sST2 in acute and chronic heart failure.

Authors	HF Setting	Patients	Mean/Median Age (Years)	Mean LVEF (%)	Follow-Up Duration	Results
Januzzi et al. [46]	AHF	593	NA	NA	1 year	sST2 concentration ≥0.20 ng/mL strongly predicted death at 1 year (HR 9.3, 95% CI 1.3–17.8; *p* = 0.03).
Zhang et al. [47]	AHF	1528	58	40	573 days	Concentrations of sST2 were elevated among patients with all-cause death and cardiac transplantation (33.6% vs. 55.6%, *p* < 0.001).
Pascual-Figal et al. [48]	AHF	107	72	47	739 days	sST2 concentrations were higher in patients who died (HR per 10 ng/mL 1.09, 95% CI 1.03–1.15, *p* = 0.005 in multivariable analysis).
Tang et al. [49]	AHF	858	66	26	180 days	Higher sST2 levels were associated with an increased mortality risk at 180 days (baseline sST2 value: HR per log increase 2.21 (95% CI 1.57–3.13); follow-up sST2 value: HR 2.64 (95% CI 1.82–3.84, both *p* < 0.001)).
Breidthardt et al. [50]	AHF	207	80	40	368 days	sST2 decreased significantly during the first 48 h in survivors compared with non-survivors, and early sST2 changes independently predicted 1-year mortality (HR 1.07 for every increase of 10%; *p* = 0.02).
Boisot et al. [51]	AHF	150	NA	NA	90 days	Patients whose sST2 values decreased by 15.5% or more during the study period had a 7% lower chance of death compared to those whose sST2 levels failed to decrease.
Aimo et al. [52]	AHF	4835	NA	NA	405 days	Both admission and discharge sST2 were predictive of all-cause death and CV death, while discharge sST2 predicted rehospitalization for HF.
Manzano-Fernández et al. [53]	AHF	447	73	46	1 year	Elevated sST2 concentrations were associated with a greater mortality risk in HFpEF (HR 1.41 per ng/mL, 95% CI 1.14–1.76, *p* = 0.002) and HFrEF (HR 1.20 per ng/mL, 95% CI 1.10–1.32, *p* < 0.001).
Sugano et al. [54]	AHF	191	76	60	445 days	sST2 concentrations were associated with all-cause death, CV death and non-CV death.
Shah et al. [55]	AHF	387	58	NA	1 year	sST2 was predictive of mortality (HR per log 2.14, 95% CI 1.37–3.38, *p* < 0.001).
Ky et al. [56]	CHF	1141	56	32	2.8 years	Patients with sST2 >36.3 ng/mL had a markedly increased risk of adverse outcomes (adjusted HR 1.9; 95% CI:1.3–2.9; *p* = 0.002).
Aimo et al. [57]	CHF	5301	66	28	5 years	sST2 independently predicted 1- and 5-year all-cause and CV deaths, and 1-,3-, 6-, and 12-month HF hospitalizations.
Emdin et al. [58]	CHF	4268	68	NA	2.4 years	The risk of all-cause death, CV death, and HF hospitalization increased by 26%, 25%, and 30%, respectively, per each doubling of sST2.
Felker et al. [59]	CHF	910	59	24	1 year	sST2 was significantly associated with death or HF hospitalization, CV death or HF hospitalization, and all-cause mortality.
Broch et al. [60]	CHF	1449	72	32	2.6 years	sST2 was significantly associated with CV death, non-fatal MI and stroke (HR per unit 1.99; 95% CI 1.68–2.36; *p* < 0.001).
Najjar et al. [61]	CHF	86	73	70	522 days	Among HFpEF, sST2 was associated with death and HF hospitalization (HR per log increase 6.62, 95% CI 1.04–42.28, *p* = 0.046).

AHF, acute heart failure; CHF, chronic heart failure; CI, confidence interval; CV, cardiovascular; HR, hazard ratio; MI, myocardial infarction.

**Table 3:** Evidence for combining sST2 and NPs in acute and chronic HF.

Authors	HF Setting	Patients	Biomarkers	Results
Januzzi et al. [46]	AHF	593	sST2 and NT-proBNP	Combination of sST2 and NT-proBNP more accurately predicted death (AUC 0.80) than the single biomarker assessment (AUC 0.72 and 0.74, respectively, both *p* < 0.001).
Zhang et al. [47]	AHF	1528	sST2 and NT-proBNP	Combination of sST2 and NT-proBNP more accurately predicted all causes of death and transplantation at 1 month (AUC 0.84) than the single biomarker assessment (AUC 0.79 for NT-proBNP and AUC 0.82 for sST2).
Tang et al. [49]	AHF	858	sST2 and NT-proBNP	Adding 48 to 72 h of follow-up sST2 to the ASCEND-HF risk model, plus follow-up NT-proBNP, correctly reclassified 15.6% of subjects for the 180-day death endpoint.
Friões et al. [77]	AHF	195	sST2 and BNP	Net reclassification index after adding BNP to sST2 concentrations was 0.70 (*p* < 0.001) in patients with HFrEF and 0.31 (*p* = 0.21) in patients with HFpEF.
Pascual-Figal et al. [48]	AHF	107	sST2, NT-proBNP and hs-TnT	For each elevated marker (from 0 to 3), an adjusted analysis suggested a tripling of the risk of death (for each elevated marker, HR 2.64, 95% CI 1.63–4.28, *p* < 0.001).
Ky et al. [56]	CHF	1141	sST2 and NT-proBNP	Combination of sST2 and NT-proBNP more accurately predicted death and cardiac transplantation (AUC 0.80) than the single biomarker assessment (AUC 0.75 and AUC 0.77, respectively). The addition of sST2 and NT-proBNP reclassified 14.9% of patients into more appropriate risk groups.
Bayes-Genis et al. [78]	CHF	891	sST2 and NT-proBNP	Patients with elevated concentrations of both sST2 and NT-proBNP had a markedly increased risk of all-cause death (HR 6.38, 95% CI 4.67–9.25, *p* < 0.001).
Pascual-Figal et al. [79]	CHF	99	sST2 and NT-proBNP	The presence of both elevated NT-proBNP and sST2 (OR 37.3, 95% CI 4.0–350; *p* = 0.002) was more predictive of SCD than evaluating each biomarker separately.

AHF, acute heart failure; AUC, area under curve; CHF, chronic heart failure; CI, confidence interval; HR, hazard ratio; OR, odds ratio; SCD, sudden cardiac death.
